# Overexpression of *TaMYC2* confers freeze tolerance by ICE-CBF-COR module in *Arabidopsis thaliana*

**DOI:** 10.3389/fpls.2022.1042889

**Published:** 2022-11-14

**Authors:** Rui Wang, Mengmeng Yu, Jingqiu Xia, Jinpu Xing, Xiaopei Fan, Qinghua Xu, Jing Cang, Da Zhang

**Affiliations:** College of Life Science, Northeast Agricultural University, Harbin, China

**Keywords:** wheat, TaMYC2, TaICE41, TaJAZ7, ICE-CBF-COR module, freeze resistance

## Abstract

Dongnongdongmai No.1 (Dn1) is one of the few winter wheat varieties that can successfully overwinter at temperatures as low as -25°C or even lower. To date, few researches were carried to identify the freeze tolerance genes in Dn1 and applied them to improve plant resistance to extreme low temperatures. The basic helix-loop-helix (bHLH) transcription factor MYC2 is a master regulator in JA signaling, which has been reported to involve in responses to mild cold stress (2°C and 7°C). We hypothesized that MYC2 might be part of the regulatory network responsible for the tolerance of Dn1 to extreme freezing temperatures. In this study, we showed that wheat *MYC2* (*TaMYC2*) was induced under both extreme low temperature (-10°C and-25°C) and JA treatments. The ICE-CBF-COR transcriptional cascade, an evolutionary conserved cold resistance pathway downstream of MYC2, was also activated in extreme low temperatures. We further showed that overexpression of any of the *MYC2* genes from Dn1 *TaMYC2A, B, D* in Arabidopsis led to enhanced freeze tolerance. The *TaMYC2* overexpression lines had less electrolyte leakage and lower malondialdehyde (MDA) content, and an increase in proline content, an increases antioxidant defences, and the enhanced expression of ICE-CBF-COR module under the freezing temperature. We further verified that TaMYC2 might function through physical interaction with TaICE41 and TaJAZ7, and that TaJAZ7 physically interacts with TaICE41. These results elucidate the molecular mechanism by which *TaMYC2* regulates cold tolerance and lay the foundation for future studies to improve cold tolerance in plants.

## Introduction

Low temperature is an abiotic stress factor that seriously affect crop yield. When plants are exposed to a low-temperature environment, the cell membrane is affected and Reactive Oxygen Species (ROS) increase, which affects the normal growth of plants and reduces the crop yield (Liang et al., 2022). Wheat is one of the most important food crops in the world (Rizwan et al., 2019). The study of cold resistance in wheat is of great significance for improving wheat yield in low temperature areas. Dn1 is the first winter wheat variety that can safely overwinter at extreme temperatures of -30°C and has a recovery rate of up to 85% in the following spring. (Peng et al., 2021; Tian et al., 2021). Therefore, choosing Dn1 as the research object will be beneficial for exploring the molecular mechanism of freeze tolerance and for improving crop varieties.

Numerous genes have been identified as being involved in key mechanisms of cold stress (Castellani and Tipton, 2015; Albertos et al., 2019; Kidokoro et al., 2022). Among these, C-repeat/DREB binding factors (CBFs) are transcription factors that play significant roles in cold stress (Hwarari et al., 2022). When plants are exposed to cold stress, *CBF* genes are rapidly induced, and CBF proteins activate downstream target cold-responsive *(COR)* genes by binding to the C-repeat (CRT)/dehydration-responsive element (Kazan, 2015; Shi et al., 2018; Peng et al., 2022) to complete the self-protection of plants under cold stress. In Arabidopsis, three CBF family members (*CBF1, CBF2*, and *CBF3*) have been detected, that are tandemly distributed on chromosome 4. To date, researchers have detected CBFs in different plants and characterized their cold-tolerance functions (Wang et al., 2017a; Hwarari et al., 2022). In addition, as an MYC-type basic helix-loop-helix (bHLH) transcription factor, the inducer of CBF expression (ICE) plays a key role in cold resistance by activating the *CBF* gene (Chinnusamy et al., 2003). The ICE-CBF-COR transcriptional cascade has been established as a major regulatory mechanism for low-temperature signaling and freezing tolerance in Arabidopsis (Chinnusamy et al., 2003), rice (Xiang et al., 2007), and tomatoes (Juan et al., 2015). CBF-mediated transcriptional network is regulated at different levels in response to cold stress. Previous reports have shown that many pivotal protein kinases and TFs can interact with ICE-CBFs and affect plant cold tolerance by positively or negatively regulating them (Zhu, 2016; An et al., 2021b). However, little information is available for the molecular mechanism of ICE-CBF-COR module in winter wheat.

Plant hormones Jasmonic acid (JA) have important effects on the low-temperature stress response. In the JA signaling pathway, the F-box protein COI1 forms an SCF-type ubiquitin protein ligase E3 with SKP1 and CULLIN1. Jasmonate ZIM-domain (JAZ) acts as a repressor protein, and in the resting state, JAZs interact with downstream TFs and inhibit JA response. In the stimulated state, bioactive JA-ILE is generated, which interacts JAZ with SCF^COI1^ and is then degraded by the 26S-proteasome to release transcription factors, and the genes regulated by transcription factors are expressed (Ruan et al., 2019; Wang et al., 2020). Low temperature stress increases JA levels in wheat (Soltész et al., 2013), rice (Du et al., 2013) and Arabidopsis (Hu et al., 2013), which are associated with increased expression of JA biosynthetic genes in rice (Du et al., 2013) and Arabidopsis (Hu et al., 2013), and decreased expression of JA catabolic genes in rice (Du et al., 2013). AtJAZ1 and AtJAZ4 can interact with AtICE1/2 and inhibit the stability of AtICE and its downstream *CBF* expression, thereby negatively regulating cold tolerance (Hu et al., 2013). In apple, JAZ1 and JAZ2 interact with BBX37, inhibit the interaction between BBX37 and ICE1, and negatively regulate *CBF* expression and cold tolerance (An et al., 2021a). These studies prove that there is a close relationship between the JA and CBF pathways. However, the relationship between JA and CBF in winter wheat under the extremely freezing temperature has not yet been reported.

*MYC2* is the first described member of the MYC family (Boter et al., 2004), which is responsible for the activation of most JA-mediated responses (Schmiesing et al., 2016; Guo et al., 2022). Some studies have demonstrated that, in Arabidopsis, *MYC2* can negatively regulate *CAT2* to participate in plant responses to salt stress (Song et al., 2021).Simultaneously, *MYC2* induced by water-spray stress promotes *bHLH19* to directly activate the *ORA47* promoter by interacting with *bHLH19*, thereby improving plant resistance (Chen et al., 2016; Van Moerkercke et al., 2019). MYC2 also interacts with ERD1 and LBD40 to improve drought tolerance in plants (Li et al., 2019; Wang et al., 2019). *MYC2* plays a critical role in cold resistance. For instance, *SlMYC2* regulates polyamine biosynthesis and thus participates in MeJA-induced cold tolerance in tomato fruits (Min et al., 2021). It has been shown that PtrMYC2 interacts with PtrBADH-I to improve glycine betaine and thus cold tolerance in *Poncirus trifoliata*. MaMYC2, a core transcription factor in the JA signaling pathway, positively regulates JA-mediated cold tolerance *via* interaction with MaICE1 (Peng et al., 2013; Zhao et al., 2013). Although MYC2 has been revealed to play an important role in plant abiotic stress response, the function of TaMYC2 in wheat under freeze stress remains unclear.

In this study, we investigated the molecular mechanism by which *TaMYC2* regulates plant freeze tolerance under extreme freezing temperature. We found that TaMYC2 plays a positive regulatory role the in freezing stress response. Overexpression of *TaMYC2* enhance the Arabidopsis freezing-resistance, including the accumulation of proline content and an increase of ROS-scavenging activity. TaMYC2 functions freezing-resistance possibly through the regulation of ICE-CBF-COR regulatory cascade. Our study demonstrated how *TaMYC2* regulates JA-mediated freeze stress through *TaICE41* in wheat and laid the foundation for improving crop tolerance to extreme freezing temperature stress in the future.

## Materials and methods

### Plant materials, growth conditions, and MeJA treatment

The winter wheat cultivar Dn1 was provided by the Northeast Agricultural University(NEAU). Dn1 was planted on 12 September 2021, in a test field on at the NEAU campus in Harbin, China. At the 2-3 leaf stage, the seedlings were divided into the following 2 groups: (1) seedlings sprayed with distilled water (control group) and (2) seedlings sprayed with 1.0 mM MeJA solution at a spray dosage of 44mL· m^-2^ (MeJA treatment group) (Zhao et al., 2019). Previous studies have shown that tillering nodes are the main components of overwintering plant (Bao et al., 2021). Tillering nodes were collected when the temperature naturally decreased to 5°C, -10°C and -25°C (average minimum temperature for 10 consecutive days in the field) (**Supplementary Figure 1**). To reduce the differences between the materials used and make the experimental results more representative, different wheat tiller nodes (approximately 1 cm) were combined into one sample (0.50 g), quickly frozen in liquid nitrogen, wrapped in tin foil, and stored in a refrigerator at -80°C (Tian et al., 2021; Liang et al., 2022).

The *Arabidopsis thaliana* ecotype Columbia (Col-0) and *Arabidopsis thaliana* mutant *myc2* (*atmyc2*) were used in this study. Among them, *atmyc2* (SALK_061267) was purchased from the Arabidopsis Information Resource (TAIR) website (http://www.arabidopsis.org/). The planting methods for *Arabidopsis thaliana* were based on previous studies (Tian et al., 2021). The incubation conditions were as follows: temperature 23°C, LED tube, photon flux density 120-150 μmol·m^-2^s^-1^, light 16h/dark 8h (Tian et al., 2021). The plates were incubated at 4°C for 3 d and then transferred to a growth chamber (temperature 23°C, LED tube, humidity 60%, photon flux density 120-150 μmol·m^-2^s^-1^, light 16h/dark 8h). For the cold acclimation treatment, 4 weeks old Arabidopsis were placed in an incubator at 4°C for 3 d and then in an incubator at -10°C for 5 h. The culture was then returned to room temperature for 7 d for observation.

### RNA extraction and real-time quantitative PCR assays

Total RNA from the wheat tillering nodes was extracted using an Ultrapure RNA Kit (CWBIO, Jiangsu, China). The obtained RNA was reverse-transcribed into cDNA using the TransScript One-Step gDNA Removal and cDNA Synthesis SuperMix (TransGen Biotech, Beijing, China). ChamQ Universal SYBR qPCR Master Mix (Vazyme, Nanjing, China) was used for real-time fluorescence quantitative PCR, according to the manufacturer’s instructions. Quantitative primers were designed using WheatOmics (http://202.194.139.32/), and are described in detail in the **Supplementary Table**. Three replicates were established for each group of samples, and *TaACTIN* and *AtACTIN* were used as the internal reference gene for qRT-PCR. For Dn1, the tillering node is an important overwintering organ (Bao et al., 2021). Therefore, the tillering nodes of Dn1 under natural cooling conditions were selected for qRT-PCR. Relative expression levels were calculated using the 2 ^-ΔΔct^ method.

### Cloning and bioinformatics analysis of *TaMYC2A, B, D*

The sequences of the *TaMYC2A, B, D* genes were obtained from WheatOmics (http://202.194.139.32/) (Gene IDs: *TraesCS1A02G193200*, *TraesCS1B02G208000* and *TraesCS1D02G196900*). PCR was used to amplify the complete DNA sequences (CDSs) of *TaMYC2A, B, D*. Primers used for PCR are listed in the **Supplementary Table**.

The nucleotide sequence of *TaMYC2A, B, D* and the amino acid sequences of TaMYC2A, B, D proteins were used to search for homologous genes and proteins in the NCBI database. The phylogenetic analysis was performed using MEGA 11. The phylogenetic trees were constructed according to the method of Liang et al. (Liang et al., 2022). The motif analysis was performed using the MEME (https://meme-suite.org/meme/tools/meme). The domain prediction was performed using NCBI Batch CD-Search (https://www.ncbi.nlm.nih.gov/Structure/bwrpsb/bwrpsb.cgi).

### Plant expression vector construction and transformation

Full-length CDSs of *TaMYC2A, B, D* were inserted into pCAMBIA1301 plant expression vectors to generate 35S:*TaMYC2A, B, D* constructs using User cloning technology (Clough and Bent, 1998). The constructed vector was then transferred into EHA105. Next, the 35S: *TaMYC2A, B, D* vectors were transformed into WT and *atmyc2* mutant to generate overexpressed (OE *TaMYC2A, B, D*) and rescued (*myc2/TaMYC2A, B, D*) plants (Clough and Bent, 1998). The screening method for homozygous transgenic Arabidopsis was based on a previous study (Tian et al., 2021).

### Subcellular localization

The full-length CDS of TaMYC2D, lacking its termination codon was inserted into the 35S: GFP vector (primers used are listed in the **Supplementary Table**). The recombinant vectors 35S: TaMYC2D *-*GFP and 35S: GFP (control) were instantaneously transformed into *Nicotiana benthamiana* using the leaf blade injection method (Liu et al., 2002). Tobacco plants were selected for each experiment, and the experiment was repeated thrice to eliminate interference from external factors. The transient expression of TaMYC2D was detected using a laser confocal microscope (TCS SP8; Leica, Germany).

### Determination of root length sensitivity to JA

The seeds of WT, *atmyc2* and transgenic Arabidopsis were poured into EP tubes, 1 mL of 75% alcohol was added, and the seeds were sterilized by shaking for approximately 1 min. The alcohol was poured out and cleaned with distilled water, and the seeds were then sterilized with 2.5% (w/v) NaClO for 5 min and cleaned with distilled water. The washed WT, *atmyc2*, and transgenic Arabidopsis seeds were placed on 1/2 MS medium for the control group and 1/2 MS medium for the experimental group containing 50 µM MeJA. The culture dish was placed vertically in a light incubator (16/8h photoperiod) and root growth was observed. After approximately 7 d, photo graphs were taken. Finally, ImageJ software was used to measure the root length.

### Determination of physiological indexes

The relative electrolyte leakage (%), malondialdehyde (MDA) content and proline content in Arabidopsis leaves were estimated according to the methods described by Lu et al, Abrahám et al. and Ma et al. (Lu et al., 2007; Abrahám et al., 2010; Ma et al., 2015).

### Histochemical staining and ROS content determination

DAB and NBT staining were performed as previously described experimental methods (Tian et al., 2021). The H_2_O_2_ and ${\text{O}}_{2}^{•-}$ contents were determined using an H_2_O_2_ and ${\text{O}}_{2}^{•-}$ content determination kit (spectrophotometric method) (KeMing Biotechnology, Suzhou, China).

### Determination of antioxidant enzyme activity

The activities of CAT and SOD were determined using a CAT and SOD determination kit (spectrophotometric method) (KeMing Biotechnology, Suzhou, China).

### Yeast two-hybrid assay

The coding regions of *TaMYC2D* and *TaJAZ7* were cloned into a pGBKT7 (BD) vector. For TaMYC2D-pGBKT7, 200ng·ml^-1^ Aureobasidin (AbA) was used to inhibit auto activation. Similarly, the coding region of *TaICE41* and *TaJAZ7* were inserted into the vector pGADT7 (AD) (the primers used are listed in the **Supplementary Table**). The recombinant vector was then transferred into AH109. Next, positive clones were screened on SD/-Trp/-Leu synthetic defect medium and protein interactions were verified on SD/-Trp/-Leu/-His/-Ade synthetic defect medium (Peng et al., 2021; Liang et al., 2022).

### Bimolecular fluorescent complimentary assay

For the BiFC assay, the full-length coding sequences of TaMYC2D and TaICE41, TaJAZ7 without their stop codons in fusion with GFP^N^ and GFP^C^, respectively, were cloned into the pCAMBIA2300-BiFC vector. Negative control was set. The constructed vector was transferred into GV3101 strain and transiently expressed in tobacco. The experiment was performed as previously described (Peng et al., 2021). Tobacco plants were selected for each experiment, and the experiment was repeated thrice to eliminate interference from external factors. The GFP signals in the leaves were observed under a laser confocal microscope (TCS SP8, Leica, Germany).

### Statistical analysis

In this study, error lines were obtained from three biological replicates. IBM SPSS Statistics 26 was used to analyze the mean values of the experimental data by one-way ANOVA analysis. The comparison method was the least significant difference (LSD), and the bar graphs with different letters indicated significant differences (p< 0.05). The bars were drawn using GraphPad prism 9.

## Results

### Exogenous application of methyl jasmonate improves the freeze resistance of wheat Dn1

**Figure 1:**
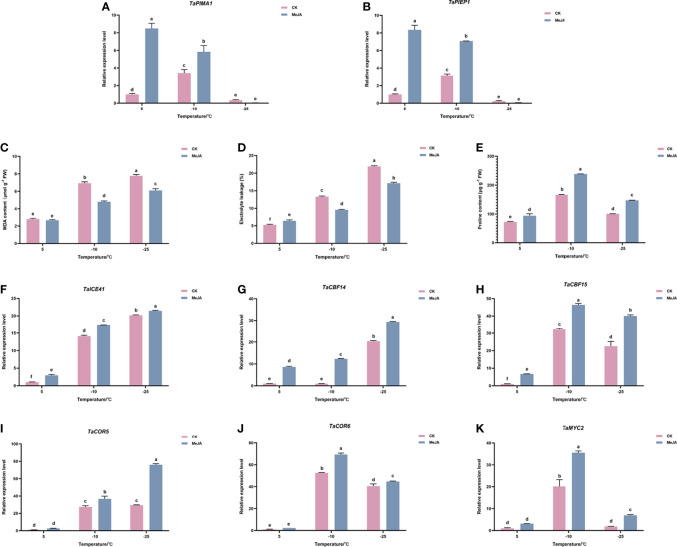
Effects of exogenous MeJA on **(A)** *TaPIEP1* expression **(B)** *TaPIMA1* expression **(C)** electronic leakage, **(D)** MDA content, **(E)** proline content, and **(F–J)** expression of *ICE-CBF-COR* cold resistance related genes. **(K)** *TaMYC2* expression of Dn1 tillering node under cold stress. The error lines were obtained from three biological replicates. The mean values of the experimental data were analyzed by one-way ANOVA analysis. The comparison method was the least significant difference (LSD), and the bar graphs with different letters indicated significant differences (*p* < 0.05).

To evaluate the effect of MeJA treatment on Dn1, we first examined the expression levels of *TaPIEP1* (pathogen-induced ERF encoding gene) and *TaPIMA1* (pathogen-induced MATE) genes, which were previously reported to be the marker genes in response to JA (Dong et al., 2010; Su et al., 2022). The results showed that at 5°C and -10°C, the expression of *TaPIEP1* and *TaPIMA1* in the MeJA treatment group was significantly higher than that in the control group (**Figure 1A, B**). This suggested that MeJA treatment can have an effect on Dn1 and has caused high expression of JA-induced genes.

We then determined the effect of MeJA on physiologic parameters of freezing resistance in wheat. As shown in **Figure 1C, D**, the electrolyte leakage and MDA content of the control and MeJA treatment group did not change significantly at 5°C; however, when the temperature continued to decrease to -10°C or even -25°C, the electrolyte leakage and MDA content of the treatment group were significantly lower than those of the control group (**Figure 1C, D**). The proline content in the treatment group was always higher than that in the control group (**Figure 1E**).

Gene expression analysis of ICE-CBF-COR module showed that inducers of CBF expression (*TaICE41*), *TaCBF* (*TaCBF14* and *TaCBF15*), and the cold-responsive genes *TaCOR* (*TaCOR5* and *TaCOR6*) increased under the freezing temperature, reaching the highest levels at -25°C. Their expressions were upregulated after MeJA treatment, which indicated that MeJA treatment promoted freeze tolerance in plants by increasing the expression of *ICE-CBF-COR* genes. (**Figure 1F–J**).

### The expression of *TaMYC2* were induced in wheat Dn1 by freezing temperature and MeJA treatment

Because MYC2 has been reported to act as an important regulator upstream of ICE-CBF-COR module, the expression of *TaMYC2* in wheat freeze resistance was detected using quantitative RT-PCR (qRT-PCR). As shown in **Figure 1K**, *TaMYC2* increased with a decrease in temperature, and the expression of *TaMYC2* peaked at -10°C, but decreased to a certain extent at -25°C, which was still greater than that at 5°C. Under cold stress, the expression of *TaMYC2* in the treatment group was greater than that in the control group, especially at -10°C, when the expression of *TaMYC2* in the treatment group was 1.75 folds than that in the control group (**Figure 1K**). These results indicated that *TaMYC2* expression is regulated by low temperature and MeJA.

Combined with the results that both freezing temperature and MeJA treatment up-regulated the expression of *ICE-CBF-COR* genes, we hypothesized that *TaMYC2* may be involved in the regulation of wheat freezing resistance through the ICE-CBF-COR pathway.

### Cloning and characterization of *TaMYC2* from wheat Dn1

Since wheat is a hexaploid plant with 3 genomes, the *TaMYC2A, B, D* sequences were obtained using WheatOmics 1.0. The *TaMYC2A, B, D* open reading frame (ORF) was isolated from the Dn1 tillering nodes based on putative sequence information available from the WheatOmics database. Sequence analysis showed that the *TaMYC2A* gene contained 2 exons and 1 intron, and *TaMYC2B, D* gene contained 1 exon. *TaMYC2A, B* encodes 693-amino-acids with an estimated molecular mass of 75.1KDa, and *TaMYC2D* encodes 695-amino-acids with an estimated molecular mass of 75.3KDa.

Phylogenetic analysis showed that *TaMYC2A, B, D* were most homologous to *ZmMYC2* in Zea mays. In contrast, the relationship between Arabidopsis *AtMYC2* and *MaMYC2* was weak (**Supplementary Figure 2**). Motif analysis showed that the TaMYC2A, B, D proteins share 10 motifs with MYC2 proteins of tomato, tobacco, Arabidopsis, and maize (**Supplementary Figure 2**). Structural domain analysis revealed that *TaMYC2A, B, D* contained the structural domain features of the plant MYC-like bHLH proteins (**Supplementary Figure 2**).

**Figure 2:**
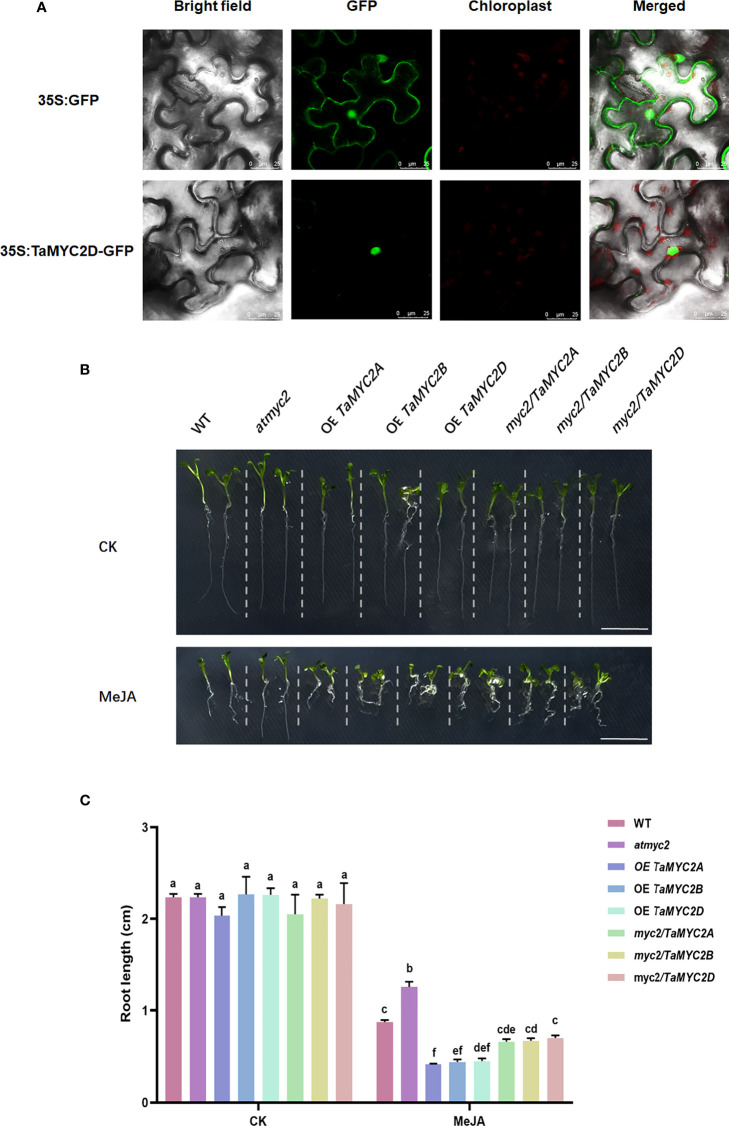
Subcellular localization of TaMYC2D in *Nicotiana benthamiana* and detection of JA sensitivity in *TaMYC2A, B, D* transgenic Arabidopsis root lengths. **(A)** Fluorescence detection of 35S: TaMYC2D-GFP fusion protein in tobacco leaves epidermal cells, and with 35S: GFP vector was used as a control (upper lane). **(B)** Root phenotype and **(C)** root length of Arabidopsis seedlings after 7 days of growth on medium with 50μM MeJA treated and control groups. The top to bottom on the right side of each bar chart corresponds to the left to right at the same treatment. The error lines were obtained from three biological replicates. The mean values of the experimental data were analyzed by one-way ANOVA analysis. The comparison method was the least significant difference (LSD), and the bar graphs with different letters indicated significant differences (*p* < 0.05).

To investigate the subcellular localization of TaMYC2, the coding sequence of *TaMYB2D* was expressed as a fusion of GFP in *Nicotiana benthamiana*. As is shown in **Figure 2A**, the fluorescence signal of 35S: GFP was detected in whole cells, whereas a strong fluorescence signal was only observed in the nucleus of 35S: TaMYC2D-GFP transformed cells, indicating that TaMYC2D is located in the nucleus.

### *TaMYC2A, B, D* can rescue the root sensitivity to JA in *Arabidopsis thaliana* myc2 mutant

Because JA signaling pathway can inhibit Arabidopsis root growth, and the myc2 mutant can affect this inhibition (Acosta et al., 2013), *TaMYC2A, B, D* were overexpressed in Arabidopsis myc2 mutant to assess their functions in JA signaling.

The results showed that there was no difference in the root growth phenotype among the lines on the control plates (**Figure 2B**). However, on the 1/2MS plate containing 50 µm MeJA, the root length of the WT was shortened by MeJA inhibition; *atmyc2* had the longest root length, followed by WT and *myc2/TaMYC2A, B, D*, and OE *TaMYC2A, B, D* had the shortest root length (**Figure 2B**). Root length measurements were consistent with the phenotypic observations. The root length of OE *TaMYC2A, B, D* was approximately 2.6 folds shorter than that of *atmyc2* (**Figure 2C**). This result demonstrates that *TaMYC2* in transgenic plants could respond to the JA signaling pathway and thus inhibit root length.

### Overexpression of *TaMYC2A, B, D* enhances the freezing tolerance of *Arabidopsis thaliana* under the extremely freezing temperature

**Figure 3:**
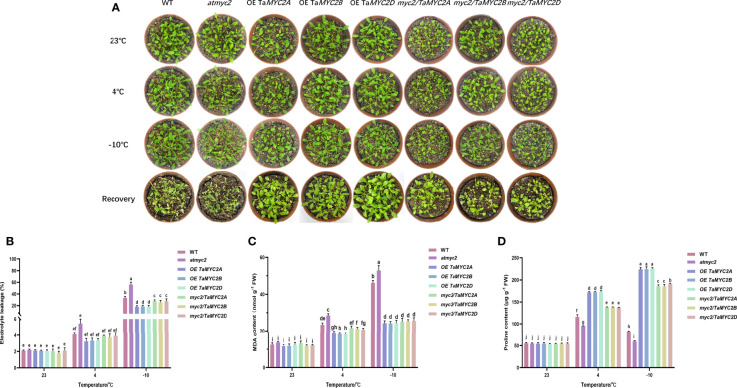
Phenotype investigation and physiological index measurement of WT, *atmyc2* mutants and transgenic plants under cold stress. **(A)** Cold resistance phenotypes of WT, *atmyc2* mutants and transgenic plants. **(B)** Electrolyte leakage, **(C)** MDA content, and **(D)** proline content of the WT, *atmyc2*, and transgenic plants. The top to bottom on the right side of each bar chart corresponds to the left to right of the same temperature. The error lines were obtained from three biological replicates. The mean values of the experimental data were analyzed by one-way ANOVA analysis. The comparison method was the least significant difference (LSD), and the bar graphs with different letters indicated significant differences (*p* < 0.05).

As shown in **Figure 3A**, there were no clear differences in the phenotypes of these Arabidopsis lines at 23°C, including growth, leaf size, and color. As the temperature decreased, the leaves gradually became slightly transparent. After recovery, it was found that most of the leaves of WT Arabidopsis were yellow, the leaves of *atmyc2* were the most yellow, and the leaves of OE *TaMYC2A, B, D.* Arabidopsis plants had almost no yellow leaves (**Figure 3A**).

For the electrolyte leakage, MDA content, and proline content of the plants, there is no significant change between WT, *atmyc2* and transgenic plants at 23°C, but when the temperature decreased to 4°C and -10°C, it was found that the electrolyte leakage and MDA of OE *TaMYC2A, B, D* Arabidopsis were lower than those of WT Arabidopsis and even lower than those of *atmyc2* plants (**Figure 3B, C**). At the same time, the proline content in OE *TaMYC2A, B, D* Arabidopsis was the highest, and *atmyc2* plants were the lowest under low-temperature stress (**Figure 3D**). This phenomenon was more obvious when the temperature was continuously reduced to -10°C.

**Figure 4:**
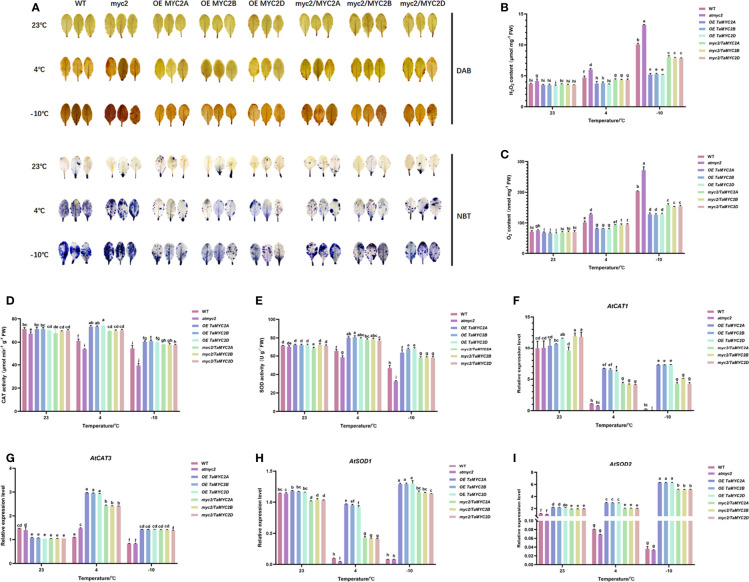
Histochemical staining and determination of ROS content and antioxidant enzymes in WT, *atmyc2* mutants, and transgenic Arabidopsis under low temperature stress. **(A)** DAB (yellow) and NBT (blue) staining of the leaves under cold stress. **(B, C)** H_2_O_2_ and ${\text{O}}_{2}^{•-}$ content determination. **(D, E)** Determination of CAT and SOD activity. Expression of **(F)** *AtCAT1*, **(G)** *AtCAT3*, **(H)** *AtSOD1*, **(I)** *AtSOD2* in WT, *atmyc2* mutants, and transgenic Arabidopsis under low temperature stress. The error lines were obtained from three biological replicates. The mean values of the experimental data were analyzed by one-way ANOVA analysis. The comparison method was the least significant difference (LSD), and the bar graphs with different letters indicated significant differences (*p* < 0.05).

Under cold stress, ROS are toxic to cells (Nadarajah, 2020). Analysis of ROS content and antioxidant enzyme activities showed that *atmyc2* plants had the most colored leaves after DAB and NBT staining, followed by WT, *myc2/TaMYC2A, B, D* Arabidopsis, and OE *TaMYC2A, B, D* Arabidopsis (**Figure 4A**). H_2_O_2_ and ${\text{O}}_{2}^{•-}$ contents in transgenic plants increased with a decrease in temperature (**Figure 4B, C**). Under the same low-temperature stress, the contents of H_2_O_2_ and ${\text{O}}_{2}^{•-}$ in OE *TaMYC2A, B, D* Arabidopsis were the lowest, while the contents of H_2_O_2_ and ${\text{O}}_{2}^{•-}$ in *atmyc2* plants were the highest. CAT and SOD activities of OE Ta*MYC2A, B, D* Arabidopsis were the strongest under low-temperature stress, followed by *myc2/TaMYC2A, B, D* Arabidopsis, and WT, whereas *atmyc2* plants had the lowest activity (**Figure 4D, E**). The qRT-PCR results of antioxidase related genes (*AtCAT1, AtCAT3, AtSOD1* and *AtSOD2*) showed that the expression levels of OE *TaMYC2A, B, D* and *myc2/TaMYC2A, B, D* were always higher than those of WT and *atmyc2* under the same low temperature stress (**Figure 4F–I**).

### Overexpression of *TaMYC2A, B, D* enhances the expression level of *ICE-CBF-COR* genes in *Arabidopsis thaliana* under the extremely freezing temperature

**Figure 5:**
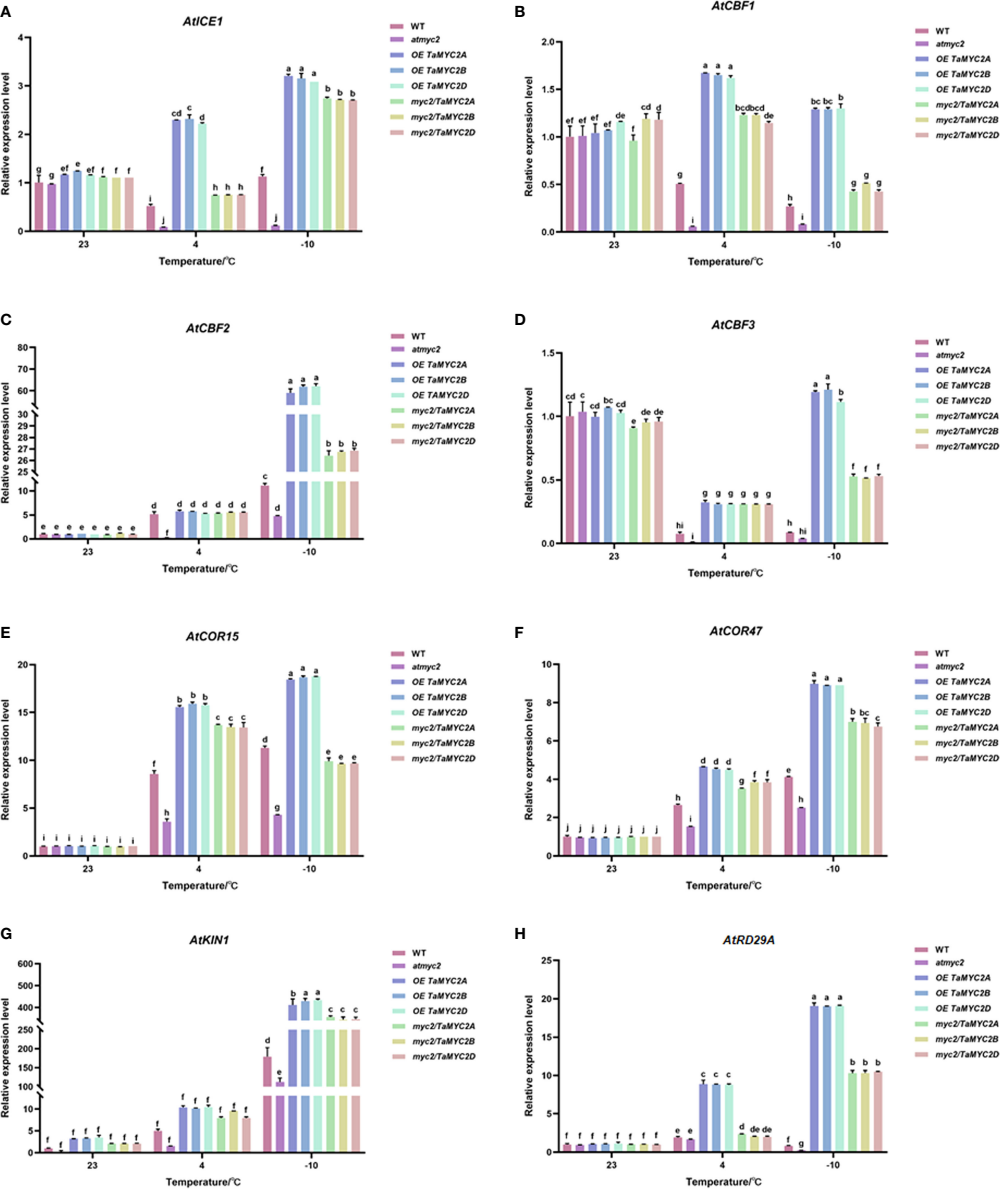
Effects of *TaMYC2* on the ICE-CBF-COR module in Arabidopsis under cold stress. Expression of **(A)** *AtICE1*, **(B)** *AtCBF1*, **(C)** *AtCBF2*, **(D)** *AtCBF3*, **(E)** *AtCOR15*, **(F)** *AtCOR47*, **(G)** *AtKIN1*, **(H)** *AtRD29A* in WT, *atmyc2* mutants, and transgenic Arabidopsis under low temperature stress. The top to bottom on the right side of each bar chart corresponds to the left to right sides of the same temperature. The error lines were obtained from three biological replicates. The mean values of the experimental data were analyzed by one-way ANOVA analysis. The comparison method was the least significant difference (LSD), and the bar graphs with different letters indicated significant differences (*p* < 0.05).

To verify whether the improved freeze tolerance of *TaMYC2A, B, D* was related to *ICE-CBF-COR* pathway, we performed qRT-PCR for *ICE-CBF-COR* genes (*AtICE1, AtCBF1, AtCBF2, AtCBF3, AtCOR15, AtCOR47, AtRD29A*, and *AtKIN1*) in transgenic Arabidopsis under freezing stress. The results showed that the expression of *ICE-CBF-COR* genes in WT, *atmyc2*, OE *TaMYC2A, B, D* and *myc2/TaMYC2A, B, D* Arabidopsis plants did not change significantly at 23°C (**Figure 5A–H**). When the temperature was reduced to 4°C, most of the *ICE-CBF-COR* genes (*AtICE1, AtCBF1, AtCBF3, AtCOR15, AtCOR47, AtKIN1*) in OE *TaMYC2A, B, D* and *myc2/TaMYC2A, B, D* transgenic Arabidopsis were significantly greater than those in WT and *atmyc2* Arabidopsis, except for *AtCBF2* and *AtRD29A* genes (**Figure 5A, B, D-F, H**). When the temperature was lowered to -10°C, the expression of *ICE-CBF-COR* genes in OE *TaMYC2A, B, D* and *myc2/TaMYC2A, B, D* Arabidopsis were more significantly different from those in WT and *atmyc2* Arabidopsis. At the same time, we found that there were no significant variations in the qRT-PCR results for *TaMYC2A, B, D*. This suggests that *TaMYC2* enhances freeze tolerance in Arabidopsis, which is associated with the activation of the ICE-CBF-COR pathway.

### TaMYC2D forms a complex with TaICE41 and TaJAZ7

**Figure 6:**
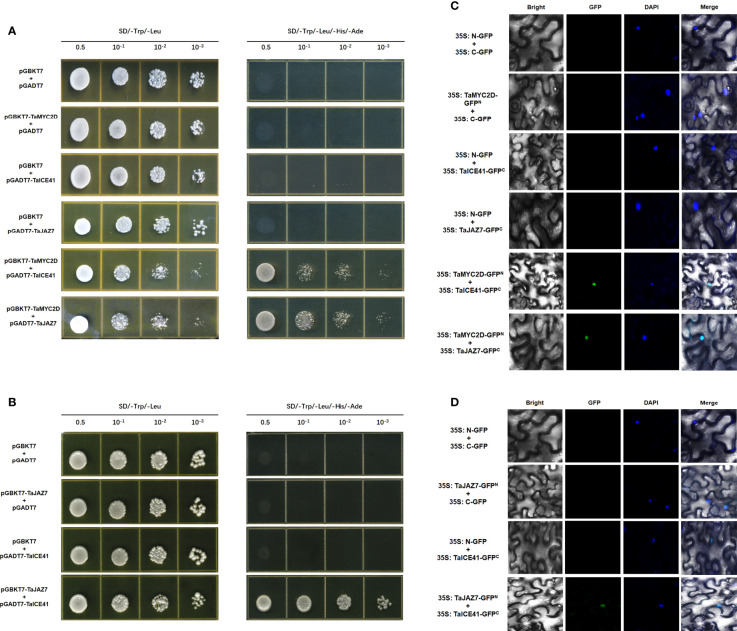
Protein interacts with protein. TaMYC2D protein physically interacts with TaICE41 and TaJAZ7 protein. **(A)** Y2H and **(C)** BiFC analysis. TaJAZ7 protein physically interacts with TaICE41 protein. **(B)** Y2H and **(D)** BiFC analysis.

The interaction between TaMYC2D, TaICE41, and TaJAZ7 were assessed using the Y2H method. The results showed that TaMYC2D interacted with TaICE41 and TaJAZ7 (**Figure 6A**), and TaJAZ7 interacted with TaICE41 (**Figure 6B**)

To confirm the interactions between these proteins, BiFC test were performed using tobacco leaves. The results showed a GFP fluorescence signal was observed in the nucleus, when TaMYC2D co-expressed with TaICE41 and TaJAZ7, individually, and when TaICE41 was co-expressed with TaJAZ7 (**Figure 6C, D**).

## Discussion

### Exogenous MeJA treatment improved freeze resistance of wheat

The adaptation of plants to the environment is the result of long-term evolution, which is regulated by many internal and external factors, such as plant hormones, light, age. For example, day length and light quality significantly affect the frost tolerance of plants (Maibam et al., 2013). Blue light receptor CRY2 enhances plant low temperature tolerance through the COP1-HY5-BBX7/8 signaling pathway (Li and Hiltbrunner, 2021). The stability of red light receptor phyB protein enhances the freeze tolerance of plants at low temperature through the CBFs interact with PIF3 (Jiang et al., 2020). In addition, the freezing tolerance was increased with plant age at the vegetative stage (Zhao et al., 2022). In this study, we mainly focus on the influence of plant hormone JA on cold resistance of wheat under the same environmental conditions. We found that exogenous MeJA treatment significantly enhanced the freezing resistance of winter wheat Dn1.

Although the role of JA and JA-mediated CBF pathways in cold stress has also been confirmed in recent years (Hu et al., 2013; An et al., 2021a), a few researches were reported on the regulation of JA in freeze resistance of wheat under extremely low temperature. In the present study, we showed that application of MeJA to winter wheat Dn1 significantly enhanced the freeze resistance under the freezing temperature (-25°C) (**Figure 1C–E**). The expression of ICE-CBF-COR cascade, a well-known cold responsive gene module, and MYC2, a master transcription factor in JA signaling pathway were also increased in Dn1 after MeJA treatment under low-temperature stress (**Figure 1F–J**). These results suggest that JA signaling and ICE-CBF-COR cascade play a critical role in freezing stress responses in plants.

### TaMYC2 activates the ICE-CBF-COR cold resistance pathway through interaction with ICE

MYC2 plays a critical role in cold stress responses in many plants. Previous studies have shown that exogenous MeJA treatment increases the expression of *MYC2* in banana (Zhao et al., 2013), tomato (Min et al., 2018) under mild-cold temperature (7°C and 2°C). In this study, we measured the changes in *TaMYC2* expression under extremely low-temperature stress treated with MeJA, and the results showed that *TaMYC2* expression increased with decreasing temperature, reaching a peak at -10°C (**Figure 1K**). Moreover, the expression of *TaMYC2* was significantly increased by MeJA treatment (**Figure 1K**). These data indicated that *TaMYC2* may be closely related to JA-mediated freeze tolerance.

TaMYC2 funtion as a key regulator in plant freezing resistance possibly through activating *ICE-CBF-COR*. Our result showed that *TaMYC2A, B, D* might activate the *ICE-CBF-COR* cold resistance pathway. This is different from previous study by Hu et al. (Hu et al., 2013), which detected the expression levels of *CBF1, CBF2*, and *CBF3* in *Arabidopsis thaliana* WT and *atmyc2* mutants at 4°C and found that there was no difference between WT and *atmyc2* mutants (Hu et al., 2013). Regardless, our study demonstrated that the cold-resistance function of *TaMYC2s* in Arabidopsis and *OE TaMYC2A, B, D*-Arabidopsis can survive under the -10°C low temperature. Therefore, *TaMYC2* can be potential genes for future genetic breeding to improve plant freezing resistance under extremely low temperature.

Most studies focused on the roles of MYC2 in activating the expression of ICE in *ICE-CBF-COR* module. To date, few studies was performed about the interaction between MYC2 and ICE in plants, except banana. Zhao et al. showed that MaMYC2 interacts with MaICE41 to participate in the cold resistance pathway of CBF at 7°C and JA pathway participates in the CBF signaling pathway through MaMYC2-MaICE1 (Zhao et al., 2013). In this study, we selected TaICE41, a sequence highly homologous to MaICE1 and AtICE1 in wheat. Considering the similarity in the domain, motif, and protein localization between TaMYC2 and MaMYC2, we hypothesized that TaMYC2 might also be linked to TaICE41. The *TaMYC2A, B, D* genes had the same effect on the freeze tolerance of transgenic Arabidopsis and the *ICE-CBF-COR* gene; therefore, we only selected TaMYC2D for the following interaction experiment. To test this hypothesis, we verified the interaction between TaMYC2 and TaICE41 by using Y2H and BiFC (**Figure 6A, C**). In addition, since *MYC2* is thought to be repressed by JAZ7 in JA signaling, we detect the interaction between TaMYC2 and TaJAZ7, which has high homology with AtJAZ1 (Wang et al., 2017b). Our result showed that there is an interaction between TaMYC2D and TaJAZ7 (**Figure 6A, C**). These results suggest that TaJAZ7 may inhibit TaMYC2 and thus affect the effect of TaMYC2 on TaICE41, which in turn affects plant freezing tolerance. JA catalyzes the degradation of JAZ by SCF^COI1^ ubiquitin ligase and 26S proteasome (Thines et al., 2007). This partly explains our previous finding that exogenous MeJA treatment increases *TaMYC2* expression.

### TaMYC2A, B, D improved cold tolerance of transgenic *Arabidopsis thaliana* through ROS metabolic

ROS are major regulatory molecules in plants and play a role in signaling events triggered by cellular metabolic disturbances and environmental stimuli (Waszczak et al., 2018). Under abiotic stress, ROS have dual functions; at high levels, they are toxic to cells, whereas appropriate levels of ROS can activate plant defense systems (Nadarajah, 2020). This study showed that the H_2_O_2_ and ${\text{O}}_{2}^{•-}$ contents of OE *TaMYC2A, B, D* Arabidopsis and *myc2/TaMYC2A, B, D* Arabidopsis were lower than those of WT and *atmyc2* under cold stress, both by chemical staining and quantitative determination. Simultaneously, the H_2_O_2_ and ${\text{O}}_{2}^{•-}$ contents in *myc2/TaMYC2A, B, D* Arabidopsis were significantly lower than those in the *atmyc2* mutant (**Figure 4B, C**). Furthermore, under cold stress, the CAT and SOD enzyme activities of the OE *TaMYC2A, B, D* Arabidopsis and *myc2/TaMYC2A, B, D* Arabidopsis were higher than those of the WT and *atmyc2* mutant plants (**Figure 4D, E**). The antioxidase related genes (*AtCAT1, AtCAT3, AtSOD1* and *AtSOD2*) showed that the expression levels of OE *TaMYC2A, B, D* and *myc2/TaMYC2A, B, D* were always higher than those of WT and *atmyc2* under the same low temperature stress (**Figure 4F–I**).These results imply that OE *TaMYC2A, B, D* Arabidopsis plants could better maintain ROS homeostasis, followed by *myc2/TaMYC2A, B, D Arabidopsis* and then WT, and the ROS scavenging ability in *atmyc2* mutants was the lowest. Thus, *TaMYC2* may improve plant freezing tolerance by participating in ROS homeostasis.

## Conclusion

**Figure 7:**
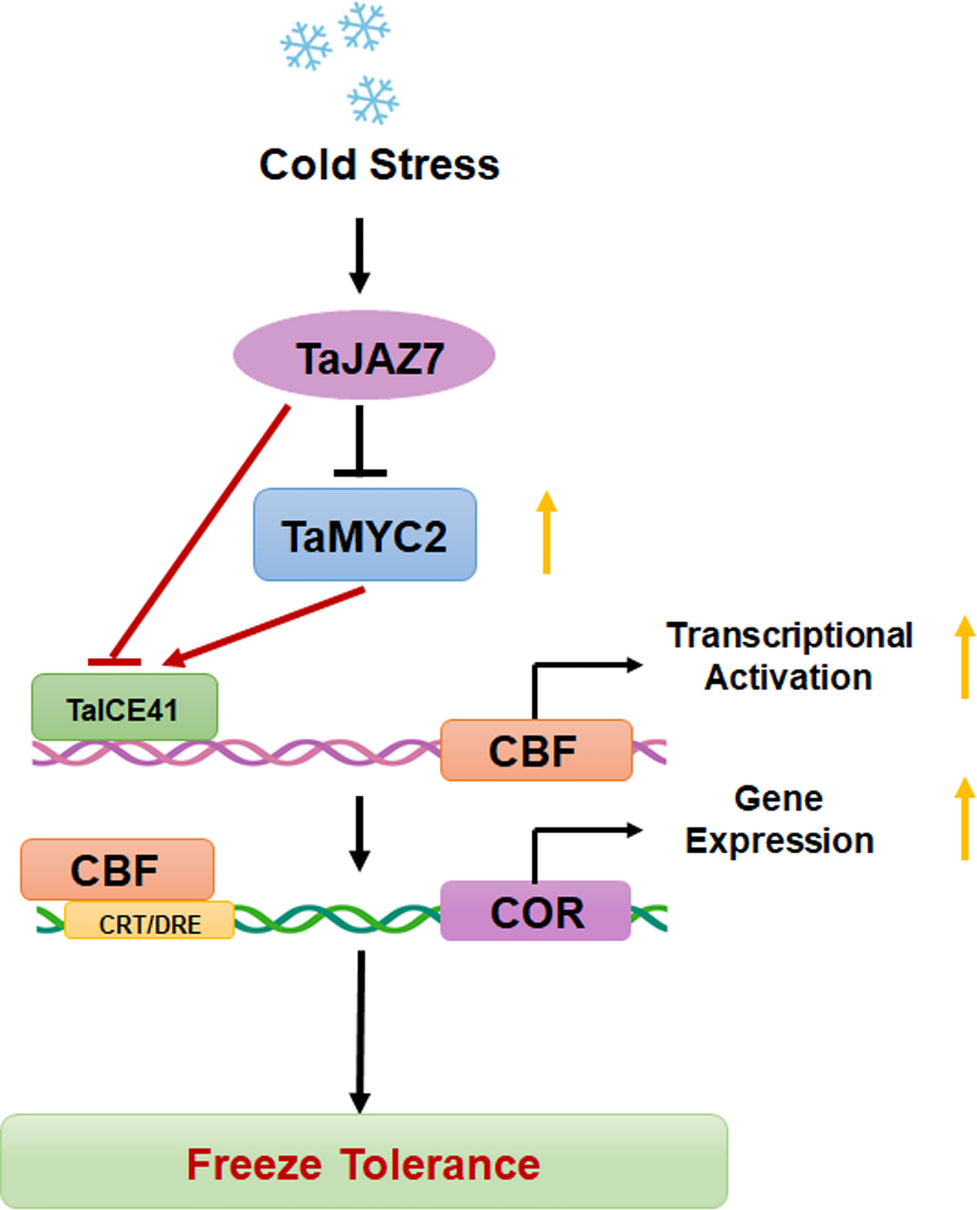
Working model diagram of the TaMYC2-TaICE41 module regulating cold stress in wheat. Yellow arrows represent the trends in gene expression.

In summary, our results demonstrate that TaMYC2 interacts with TaICE41 to activate the downstream CBF-COR pathway, thereby improving freeze tolerance in plants (**Figure 7**). Moreover, TaJAZ7 may interact with TaMYC2 to inhibit the transcriptional activity of TaICE41 or directly interfere with TaICE41 to inhibit its expression of TaICE41, thus negatively regulating freeze tolerance. These findings provided insights into the improvement of freeze tolerance in wheat, reveal the relationship between the JA and CBF pathways, and demonstrate the positive role of *TaMYC2* in freeze tolerance.

## Data availability statement

The original contributions presented in the study are included in the article/**Supplementary Material**. Further inquiries can be directed to the corresponding author.

## Author contributions

RW completed the experiments, data analysis and drafted the manuscript. MY, JqX helped in assisting with the experiments. JpX, XF, QX and JC participated in revising articles. DZ conceived and designed the study, obtained financial support, provided the study material and revised the manuscript. All authors contributed to the article and approved the submitted version.

## Funding

This work was supported by National Natural Science Foundation of China (31701348) and Natural Science Foundation of Heilongjiang Province (LH2021C022).

## Acknowledgments

We sincerely appreciate the help of Professor Yunde Zhao (University of California, San Diego), Associate Researcher Yuying Wang (Institute of Botany, Chinese Academy of Sciences, China) and Associate Professor Libo Wang (Northeast Agricultural University, China) in the revision of the manuscript. We would like to thank KetengEdit (www.ketengedit.com) for its linguistic assistance during the preparation of this manuscript.

## Conflict of interest

The authors declare that the research was conducted in the absence of any commercial or financial relationships that could be construed as a potential conflict of interest.

## Publisher’s note

All claims expressed in this article are solely those of the authors and do not necessarily represent those of their affiliated organizations, or those of the publisher, the editors and the reviewers. Any product that may be evaluated in this article, or claim that may be made by its manufacturer, is not guaranteed or endorsed by the publisher.
