# Supplementary material for: Overexpression of TaMYC2 confers freeze tolerance by ICE-CBF-COR module in Arabidopsis thaliana
Source: Front Plant Sci. 2022 Nov 14;13:1042889. doi: 10.3389/fpls.2022.1042889 (PMC9710523; doi:10.3389/fpls.2022.1042889)
Supplement: Supplementary file 1 [file DataSheet_1.docx]

Supplementary Material

# Supplementary Figure 1


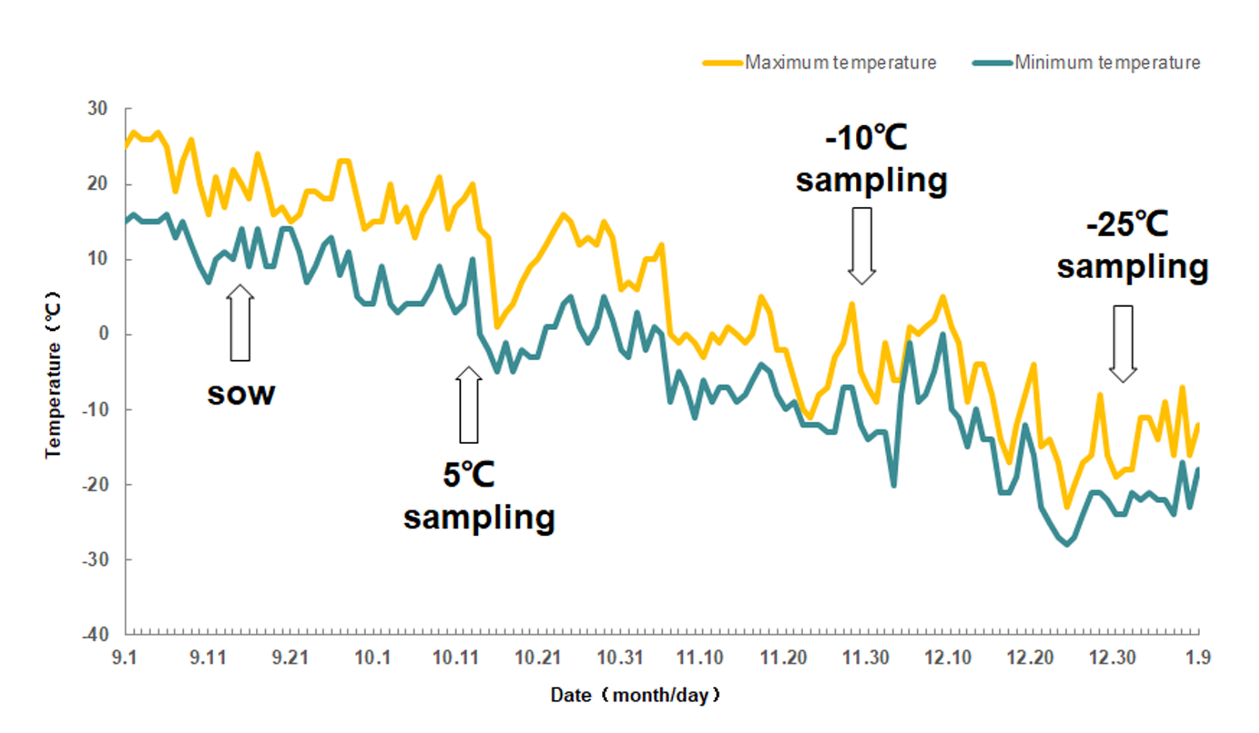


**Supplementary Figure 1.** Temperature change trend of Dn1 from sowing to sampling. The orange line represents the highest temperature of the day, and the green line represents the lowest temperature of the day.

# Supplementary Figure 2


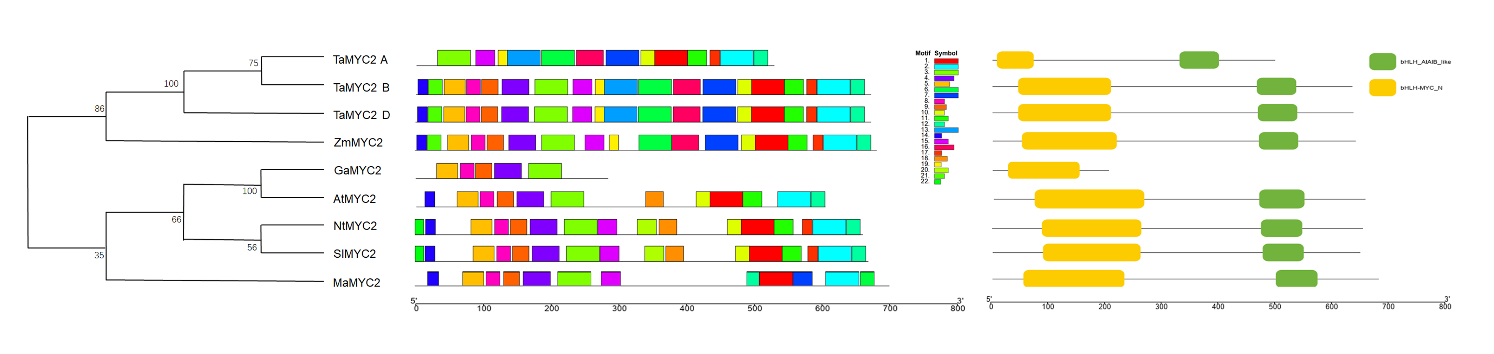
**Supplementary Figure 2.** Biological information analysis of *TaMYC2A, B, D.* Phylogenetic reconstruction using the neighbor-joining method (Jones-Talor-Thornton model) of TaMYC2A, B, D (XP_044328981.1, XP_044402249.1, XP_044449661.1) and several highly homologous MYC2 proteins, including ZmMYC2 (QDM55339.1), GaMYC2 (XP_006601141.1), AtMYC2 (NP_174541.1), NtMYC2 (NP_001312960.1), SlMYC2 (NP_001311412.1), MaMYC2 (XP_009388411). The first two letters of the sequence name represented initials of Latin name of species from which the sequence originates. Motif analysis of TaMYC2A, B, D and homologous sequences is shown in the middle, and domain analysis is shown on the far right.
